# Perceptions of Existing Wearable Robotic Devices for Upper Extremity and Suggestions for Their Development: Findings From Therapists and People With Stroke

**DOI:** 10.2196/rehab.9535

**Published:** 2018-05-15

**Authors:** Ahmed Elnady, W Ben Mortenson, Carlo Menon

**Affiliations:** ^1^ Menrva Research Group School of Engineering Science Simon Fraser University Burnaby, BC Canada; ^2^ Department of Occupational Science and Occupational Therapy Faculty of Medicine University of British Columbia Vancouver, BC Canada; ^3^ International Collaboration on Repair Discoveries Vancouver, BC Canada; ^4^ GF Strong Rehabilitation Research Program Vancouver, BC Canada; ^5^ Menrva Research Group School of Mechatronic Systems and Engineering Science Simon Fraser University Surrey, BC Canada

**Keywords:** qualitative research, focus group, wearable devices, rehabilitation, upper extremity

## Abstract

**Background:**

Advances in wearable robotic technologies have increased the potential of these devices for rehabilitation and as assistive devices. However, the utilization of these devices is still limited and there are questions regarding how well these devices address users’ (therapists and patients) needs.

**Objective:**

The aims of this study were to (1) describe users’ perceptions about existing wearable robotic devices for the upper extremity; (2) identify if there is a need to develop new devices for the upper extremity and the desired features; and (3) explore obstacles that would influence the utilization of these new devices.

**Methods:**

Focus groups were held to collect data. Data were analyzed thematically.

**Results:**

A total of 16 participants took part in the focus group discussions. Our analysis identified three main themes: (1) “They exist, but...” described participants’ perceptions about existing devices for upper extremity; (2) “Indeed, we need more, can we have it all?” reflected participants’ desire to have new devices for the upper extremity and revealed heterogeneity among different participants; and (3) “Bumps on the road” identified challenges that the participants felt needed to be taken into consideration during the development of these devices.

**Conclusions:**

This study resonates with previous research that has highlighted the importance of involving end users in the design process. The study suggests that having a single solution for stroke rehabilitation or assistance could be challenging or even impossible, and thus, engineers should clearly identify the targeted stroke population needs before the design of any device for the upper extremity.

## Introduction

Traditional, hospital-based stroke rehabilitation can be labor-intensive and expensive. In the United States alone, the direct and indirect cost for stroke rehabilitation is about US $36.5 billion per year [1]. From 2012 to 2030, the total direct annual stroke-related medical costs are expected to increase from US $71.55 billion to US $184.13 billion [2]. Furthermore, outcomes from rehabilitation are inconsistent across individuals, and recovery is hard to predict. Given these uncertainties, numerous technological approaches have been tested in an effort to improve rehabilitation outcomes and reduce the cost of stroke rehabilitation [3]. In recent years, interest has grown in the use of wearable robotic devices (ie, devices worn by human operators, whether to supplement the function of a limb or to replace it and thus enhance a person’s motion or physical abilities [4]) that aim to restore mobility in stroke population [5-16]. However, many of these devices have been developed from a technology-centered perspective, where engineers develop systems needed to provide upper extremity rehabilitation or assistance with little user input [17]. Moreover, designers are either unaware of the needs of users with different capabilities, or do not know how to accommodate their needs into the design cycle [18]. The lack of involvement of end users in the design process results in a failure to gain users’ acceptance and approval [19].

Given the multiple factors that affect a user’s decisions to use or adopt different types of devices and technologies in the clinical practice, it has been recommended that users should be involved throughout the design process; this approach is known as user-centered design. This approach is intended to help designers identify relevant aspects and different factors that should inform their design choices [20]. User-centered design has emerged in the last 30 years as an alternative approach to traditional engineering effort [21]. The purpose of user-centered design approach is to serve the user and not just the use of a specific technology. Additionally, it is an iterative process with an ultimate goal to develop usable systems and thus prompt their clinical utilization [22]. Unfortunately, little research has been conducted to explore and understand users’ perceptions and their viewpoints regarding these devices and technologies [23-26].

Given the uncertainties about the utility of existing devices for the upper extremities and the desire to develop better ones, we conducted a study with three main objectives:

1. Explore the users’ perceptions of existing devices and technologies for upper extremities and thus identify reasons that would affect whether they would use or not use these devices.
2. Investigate whether there is a need to develop new devices.
3. Identify different factors that would limit the utilization of any future devices for the upper extremities.

## Methods

### Setting, Participants, and Recruitment

The exploratory nature of this study lends itself to qualitative methods. Focus group discussions were the primary method of data collection for this study [27]. Focus groups have advantages over other data collection tools such as one-to-one interviews and surveys, as they accommodate large number of people having common interests in a specific topic [28] and enable the collection of information in a short time [29]. We have reported the study using the consolidated criteria for reporting qualitative research [30]. The study was approved by local universities and health authorities ethical review boards (REB: # 2012s0527, BREB #: H16-01085, and VCHRI: # V16-01085).

The study took place in the province of British Columbia, Canada. In this jurisdiction, there is very limited public funding available for assistive technologies (eg, disability benefits or basic medical disability plan), so most people need to rely on personal finances or extended health insurance benefits to obtain these devices [31]. Two groups of participants were invited to participate in the study. The first group was for people with stroke who had ongoing upper limb mobility problems and had experience with or interest in robotic devices. The second group was for occupational therapists and physical therapists who had at least 1 year of professional experience working with either seniors or people with stroke or were interested in robotic devices. Participants with stroke were recruited through distribution of advertisements to community centers and local supportive groups (eg, local stroke clubs). Therapists were recruited through email distribution of a letter of initial contact and posted advertisements. All participants provided informed consent. The second author knew some of the therapists who participated in the study casually as members of the same discipline.

### Data Collection

The second author, an occupational therapist who has extensive experience with individuals with neurological disorders and focus group facilitation, moderated the focus groups. During focus group interviews, participants were led through a series of questions following an interview guide that was developed after extensive discussions and review with qualitative research experts with experience in conducting focus groups. The focus group interview guide went through multiple revision cycles to make sure that the questions would be understandable by the participants (Multimedia Appendix 1). Questions probed the participants’ experiences and views of current therapeutic technologies and devices used for upper extremity, desirable features for future technology designed to rehabilitate the upper limb, and perceived barriers to use of technologies and robotic devices.

The study was conducted in a local rehabilitation center where discussions included the participants and the researchers only. At the beginning of each group, the moderator introduced the purpose of the focus group, the ground rules, the process, and the objectives of the discussion. During the introduction, the moderator reiterated the purpose of the study and encouraged an open climate for all participants to express their opinions freely. The moderator facilitated the discussion to allow the participants to enrich the conversation through interactions with each other. The first author took field notes to offer an additional perspective of the focus group findings and to provide a nuanced context of each group. The focus groups were audiorecorded and later transcribed verbatim by a research assistant. Both the first and second authors reviewed all the transcriptions to confirm the content and to identify any missing data or any discrepancies. Transcripts identified participants and researchers by numbers so that perceptions or contributions of everyone could be tracked anonymously. To further protect participants anonymity, all proper nouns (describing places or other people) were replaced with pseudonyms, and we limited the amount of personal information revealed about each participant, and we did not report quotes that might enable the participant’s identity to be easily inferred (eg, if the situation or event was very unique).

### Data Analysis

Transcripts and filed notes were analyzed thematically through a process outlined by Braun and Clarke [32]. This involved becoming familiar with the data, generating initial codes, searching for themes, reviewing themes, and defining and naming themes. The first two authors used Microsoft Excel to initially code interview transcripts, observation notes, and debriefing, following data collection sessions independently and then worked collaboratively to develop an initial coding guide, which was then applied to all of the data and eventually amalgamated into subthemes and themes.

We used two different strategies to promote trustworthiness: triangulation and reflexivity. Triangulation of participants involved the inclusion of people with stroke and clinicians. There was also triangulation of researchers as noted above. Field observation notes and debriefing following data collection sessions between the first two authors were used to facilitate reflexivity [33]. We were interested in developing new devices for upper extremity, so that may have prejudiced the researchers against existing devices. To guard against this, we tried to probe for positive features of existing technologies.

## Results

We conducted four focus groups from September 2016 to October 2016: one for people with stroke and three for therapists. The focus group for people with stroke included 8 participants and lasted for 90 min. The three focus groups for therapists included 8 participants (2-3 each), and each lasted for almost 45 min. Tables 1 and 2 show the demographic characteristics of people with stroke and therapists who joined the focus groups interviews, respectively. Our analysis of the interview data and field notes identified three main themes and 11 subthemes as described in Textbox 1 and detailed below.

**Table 1:** Demographic characteristics of participants (people with stroke, n=8).

Characteristics		n (%)
**Gender**		
	Female	1 (13)
	Male	7 (87)
**Age in years**		
	50-60	1 (13)
	61-70	5 (62)
	>70	2 (25)
**Stroke duration in years**		
	<5	1 (13)
	5-10	3 (37)
	>10	4 (50)
**Handedness**		
	Right	8 (100)
**Affected hand**		
	Right	3 (38)
	Left	5 (62)

**Table 2:** Demographic characteristics of participants (therapists, n=8, all females).

Characteristics		n (%)
**Age in years**		
	30-40	4 (50)
	40-50	2 (25)
	50-60	2 (25)
**Profession**		
	Physiotherapist	4 (50)
	Occupational therapist	2 (25)
	Rehabilitation assistant	2 (25)
**Professional experience in years**		
	<5	1 (13)
	5-10	2 (25)
	11-15	3 (36)
	16-20	1 (13)
	>20	1 (13)

**Summary of main themes and subthemes.**

1. They exist, but...
  - Existing devices and technologies
  - Cost-effectiveness
  - Doubts on efficiency
  - Compromise the independence
2. Indeed, we need more. Can we have it all?
  - Assistance vs rehabilitation
  - Distal vs proximal
  - Portability vs complexity
  - Activation and motivation
3. Bumps on the road
  - Single solution is challenging
  - Ensure accessibility
  - Setup time and learning curve

### Theme 1: “They Exist, but...”

The first theme described participants’ experience with existing devices and technologies for upper extremity. Subthemes described participants’ knowledge of existing devices and factors limiting their utilization.

All participants had knowledge about existing passive or nonrobotic devices for upper extremity. For example, one participant with stroke indicated that sometimes he used slings for stabilization. Another participant with stroke mentioned a device used for wrist and fingers extension to decrease spasticity:

The [Saebo] is a manual device for the hand with springs on it, looks like a glove. After exercising the fingers, turn the fingers on.

A therapist described using passive devices to decrease spasticity, maintain range of motion, and to avoid subluxation of the shoulder:

We use resting hand splints with our stroke clients..., for...night time, helping to...maintain range and manage their spasticity and we have a couple different shoulder supports for like hemiplegic shoulders, subluxation of the shoulder.

Thus, these devices were widely known and used for different joints and different reasons.

Available active devices for upper extremity were not as popular as the passive ones. Only one stroke participant mentioned a commercially available active device for upper extremity. He stated the following:

The [Bioness] opens my hand and closes it. My hand naturally tells itself [to do this]. So this helps my hand. It’s electronic.

Participants mentioned that the utilization of existing devices was limited because of many issues. Participants went through some barriers such as the initial cost, doubts on the efficacy of such devices, interfering with their activities, and compromising their independence.

All participants reported that the initial cost of any device would affect their decision to adopt it (as for therapists) or to use it (as for people with stroke). For example, one participant with stroke considered the cost of a device he had before was overpriced. Another example is a therapist who described a device developed a few years ago and was unaffordable for her clients. She stated the following:

I can recall an in service with a splint a number of years ago when they came in and showed us I would call it, sort of robotic like, where it helps them to do a certain pattern of movement. But quite expensive, we didn’t really think...our clients would be able to afford it.

The cost was an important consideration not only for patients but also for therapists who could potentially act as gatekeepers to these devices. Thus, a high cost could potentially hinder the utilization of any device for upper extremity rehabilitation.

Limited evidence of clinical utility of existing upper extremity devices was considered as a barrier. For example, one participant with stroke shared his experience with a company that sells a device for the upper extremity. He stated the following:

By the way, I talked to [a company] last week...They still have not written a manual for recovery,...they still do not have clinical trials.

Another participant with stroke questioned the efficacy of rehabilitation in general. He stated the following:

I am completely paralyzed on the left side and with probably no chance to recover, I don’t know what to do but robotic devices, I see this stuff on the computer and YouTube. But without [the] brain active, doesn’t do any good.

These doubts about the efficacy of existing devices for upper extremity rehabilitation were considered a barrier to their utilization.

The size of upper extremity devices was a barrier, given a perception that available devices are either bulky or uncomfortable. A therapist indicated that the size of many devices was annoying for her clients, as she wanted her clients to use these devices while they are sleeping; however, there was an issue with adherence. She stated the following:

A lot of people say they don’t like to wear the splint [ie, a passive device to maintain wrist or hand posture and to decrease spasticity] because it’s bulky...People don’t wear these devices because the Velcro catches on their sheets.

Thus, the size of upper extremity devices was a limiting factor that might prevent some people from using them.

Participants were concerned about the long-term implications of device use. A stroke participant shared his experience from the acute phase of his injury and suggested that relying on assistive devices would have compromised his independence. He stated the following:

I had my stroke 9 years ago hemiplegic...I could not eat, and I could not talk... was completely paralyzed on the right side...From the beginning; I was against devices to help me, because I did not want to rely [on] high tech.

Another stroke participant shared similar experience after being discharged from the hospital, as he refused to rely on wheelchair as an assistive device. He stated the following:

When I came home back [released from the hospital] my kid brought me a wheelchair,...I said never, I would get into one of these,...I am walking around for 6 months now.

Thus, concerns about the long-term impact of these devices on participants represented a disincentive for their use among some participants.

### Theme 2: “Indeed, we Need More. Can we Have it all?”

All participants identified a need to develop new devices for upper extremity. Participants with stroke mentioned personal reasons to develop new devices, as they were keen to have assistive devices to help in daily life activities (such as word processing or typing, cooking, drinking, etc). One participant with stroke indicated that she wanted devices to help in her kitchen:

Try cooking with it,...you’re holding onto a bowl and not holding on very well...You’re stirring well in it but it’s going all over the place on the floor.

In contrast, therapists’ needs were more diverse and covered larger spectrum of potential users. For example, a therapist wanted to have devices that would complement traditional therapy, such as a preparation for rehabilitation sessions to reduce the traditional therapy sessions as an adjunct to the therapy. He stated the following:

You know we’re talking active rehab, prepping it for therapy sessions or for use in the therapy session. Or potentially you could still say it was part of your therapy session.

Thus, the needs were different; participants with stroke were in the favor of assistive devices, whereas therapists demonstrated a preference for therapeutic devices.

All participants with stroke suggested developing new devices for hand and fingers. To illustrate this need, one participant with stroke demonstrated his inability to open a bottle of water without spilling the contents. Two occupational therapists supported the need to have new devices for distal control as a motivation of active engagement and for functional training:

I think there would be pros to getting the distal because maybe if they [patients]start to activate their hand more, then we’ll actually see better proximal, I think that the research shows we don’t just lift our arms for the sake of lifting our arm[s], I mean you’re always moving your upper limbs for a purpose.

In contrast, 4 physiotherapists suggested having new devices for proximal control and stabilization; such devices would help decrease spasticity and the pain. A physiotherapist shared her thoughts about which joints considered important for upper extremity rehabilitation. She stated the following:

I think by the end of the day, the stroke will be with proximal joints more. They have a lot of pain in the shoulder because they don’t move [or] do anything with those joints.

Thus, despite the perceived need to develop new devices, participants were not unanimous about which part of the upper limb should have the priority when designing new devices.

Participants had different opinions regarding the portability and ease of donning devices for upper extremity. All participants with stroke wanted new devices to be wearable and portable, like a garment. A participant with stroke emphasized the importance of having simple devices:

Ease of insertion, you know for some people they only have one arm, you don’t want something really complex, you know, get it on get it off easily.

Therapist participants were in the favor of having comprehensive devices (ie, larger devices that could work across multiple joints) for the upper extremity. A therapist participant acknowledged that comprehensive devices would be more complicated; however, they would accommodate a broader spectrum of movements for functional training:

If you’re going to have something as a multi-joint, that would be awesome from a reach pattern perspective but then the complexity of the device, could be [too] much.

Thus, portability and easiness of donning devices for upper extremity were critical issues for stroke participants, as they could facilitate their independence; they were less critical issues for therapists.

Participants had different opinions regarding which type of signals would be the best to control or trigger devices for the upper extremity. On one hand, there was considerable variation in the methods of activation preferred by participants with stroke, and no one approach dominated. A stroke participant suggested a novel method to control a wearable hand device only by looking at the device:

I want to be able to just look at the hand and put those two fingers together.

On the other hand, therapists had more specific suggestions about signals that could be used to control or trigger these devices for distal or proximal joints. For example, a therapist suggested using biosignals to activate these devices to augment relearning movement patterns:

Brain activity would be cool, actually having them [patients] like think about the movement; it seems smart for somebody who had a stroke, which would be a good idea. EMG [electromyography] maybe if they’re showing small amounts of muscle activity, so then they [patients] might then back learn the pattern more, and with that help for further activation.

One therapist shared her experience with an exoskeleton used for lower extremity. She suggested having new devices that provide active engagement and thus provide more motivation. She stated the following:

You can see how they get really happy when they can do something with the help of something, like for example, when I see the clients working with the robotic thing for walking, hope comes, so they can be more motivated to do it.

Thus, developing devices that would provide active training may have positive psychological impacts in that success in training might motivate patients to practice more.

### Theme 3: Bumps on the Road

In addition to the issues participants perceived above about existing devices for the upper extremity, participants identified other issues and concerns that would hinder the utilization of any newly developed devices. These concerns varied from the difficulty finding a single design that would fit every one, users’ should be able to have the accessibility to such new devices, and finally, the way to set up and use these device should be as simple as possible.

All participants reflected that finding a single design is challenging, as it is difficult to identify a universal design that would work for all users. This concern was illustrated by one participant with stroke, who stated the following:

Everyone has their own situation...We’re talking about different things for different situations...there isn’t one size fits all.

Likewise, one therapist indicated that it is not clear which population would most likely use a robotic device, as there are many parameters involved, and it is difficult to decide on which joint should be the focus as it depends on client needs:

Yeah it is very client dependent. If I try to think of the spectrum of my clients, it would be very difficult to say one.

Two stroke participants reported that sometimes there were some tools or devices that would benefit them; however, they did not get access to these tools. One participant with stroke explained the following:

Access to the tool you’re using [...] might be an issue. They are not located anywhere you can use them. [People] might know about new devices or technologies; however, they are not available to every patient to use them.

Likewise, three therapists identified a concern about accessibility from a different point of view. One therapist illustrated that without financial support from a third party (eg, governments and insurance companies), the accessibility for any new developed devices would be limited:

I think without [...] extended benefits, without insurance companies maybe buying into that, I think [only] relatively small grouping of clients would be able to afford it.

Thus, having the accessibility to the right tools for rehabilitation or assistance was one of the concerns that would limit the utilization of any new device.

All therapists reported that the setup time was a concern when adopting new devices. A therapist reported that the setup time should not be more than 10 min:

Oh gosh no, I would say 10 minutes max for me...and then every 6 months, you still [got to] be able to do it in 10 minutes.

Another therapist acknowledged that using new devices is a learning process:

I think something with the exoskeleton [for the lower extremity] I noticed for initially, I know it takes some learning and it gets a little bit better but initially they [users] seem to perceptually have a really hard time with what exactly is going on in the exoskeleton.

Thus, setup time or complicated training requirements could limit adoption of new devices.

## Discussion

### Principal Findings

The objectives of this study were to explore users’ perceptions about existing wearable robotic devices for upper extremity, to identify if there was a need to design and develop new devices for upper extremity, and to describe different factors that might affect the design of new devices and thus the required features for these devices. Earlier studies have investigated user’s perception regarding specific robotic devices for upper extremity [34]; however, to the best of our knowledge, this is the first study to explore users’ perceptions regarding wearable robotic devices for the upper extremity generally, rather than being limited to a specific device. The study findings describe the perspectives of therapists and people with stroke who had previous experience with different upper extremity devices.

### Theme 1: “They Exist, but...”

The awareness that participants had about assistive technologies or devices is not surprising given previous research by Hughes et al [35] that found 92% of health care professionals had accessed information on assistive technologies, with 59% of them using assistive technologies or devices in their clinical practice, whereas 41% of patients and care givers had accessed information, with 44% of them using assistive technologies or devices. People with stroke showed more knowledge of existing devices for assistance over therapeutic devices, as their main goal is to perform their daily life activities independently. Therapists showed more knowledge of existing therapeutic devices, as their main goal is to help people with neurological disorders to recover if possible. Although there is little evidence that passive devices (eg, splints and slings) improve motor function, reduce spasticity, or prevent contractures in the upper extremity [36], participants indicated that these interventions are still relatively common and had limited awareness of active devices for upper extremity, which have been recommended as an alternative [37]. The popularity of passive devices over active devices for the upper extremity reflects a tension between research evidence and the clinical practice, which appears ongoing as reported by Hughes et al [35].

The acceptability of novel interventions such as robotic devices requires careful weighing of the perceived benefits with the potential costs, which is described as the cost-effectiveness trade-off [38]. Currently, there is a critical need for more experimental research that provides evidence about the efficacy of these devices [32] and better information about actual costs involved. For example, although participants in this study were concerned about the cost of these interventions, a study by Wagner et al [31] found no statistical difference between robotic rehabilitation cost and usual care cost. Ultimately, without a better understanding of the effectiveness of these devices and their cost to implement into practice, people with stroke and clinicians who work with them will be unable to make accurate determinations of their cost-effectiveness.

Concerns about not regaining functional independence is a barrier that may limit the utilization of wearable robotic devices for the upper extremity. Although there is controversy about how independence should be defined [38,39], stroke participants in our study suggested depending on assistive devices would be stigmatizing. This finding is congruent with previous research done by Silvers [40] that concluded that people feel stigmatized by devices that signal loss of function. Similarly, Luborsky indicated that people with stroke may not choose to adopt new technologies that allow independent mobility if this makes them feel more visibly disabled [41].

### Theme 2: “Indeed, we Need More. Can we Have it all?”

This study reveals the existence of a strong desire to develop new devices for upper extremities. Interestingly, each set of participants (eg, people with stroke and therapists.) had their own reasons to have new devices for upper extremity. In general, therapists would like to have devices that complement the traditional therapy; this finding supports the study carried out by Hughes et al [35] where the authors concluded that robot-assisted movement therapy should only be used as an adjunct to conventional therapy to minimize unwanted compensatory movements. Despite recent efforts to reduce compensatory movements using automated feedback, Valdés et al [42], our finding emphasizes concerns about the unsupervised use of these devices by therapists, which may also reflect fears that robotic devices could reduce their role during rehabilitation.

Another tension that emerged in this study was related to concerns about portability vs complexity. People with stroke wanted these devices to be as simple as possible and allow users to don and doff them without assistance. Research conducted by Colleen O'Brien Cherry et al [43] was congruent with our finding that users (people with stroke) wanted to have a device that is easy to wear and to use. The preference for therapists for a more complex device that covers a broader spectrum of patients is understandable given the heterogeneous populations they serve.

Our findings emphasize a desire for active devices that provide motivation. Acknowledging that active devices required control signals to be actuated, people with stroke suggested having simple control mechanisms (eg, the device could be triggered and moved by visual cues). Unlike verbal or auditory signals, this mechanism would draw less public attention to the user. In contrast, because the therapists were more interested in neuroplasticity augmentation, they preferred to use biosignals such as electroencephalography and electromyography to control such active devices. The simplicity of the active assistive devices that people with stroke are asking for might reflect concerns about the likeliness of recovery; thus, they may want devices to help in their daily activities regardless of any recovery goals. This variability in goal setting is congruent with previous studies by Lawler et al [44] and Dowswell at al [45] that concluded that recovery goals are relative, variable, and individually based.

### Theme 3: Bumps on the Road

In this study, some barriers were identified that would hamper the development of new devices for upper extremity and could potentially hinder their adoption. The heterogeneity of users represents a challenge for device development. Although the heterogeneity among the participants with stroke might be anticipated given that their needs and expectations from the assistive devices are diverse, the existence of such heterogeneity among occupational and physical therapists is interesting. These differences could be related to the different role they have in rehabilitation settings, as there may be more of a focus on hand function among occupational therapists [46,47]. Given these heterogeneities, finding a single solution that is accepted by the majority of the users’ may not be possible.

The ease of access to commercially available technologies or technologies under development is a crucial barrier. Limited accessibility to available resources for rehabilitation or assistance technologies would slow down their adoption. This finding is congruent with a study by Hughes et al [35] that concluded that lack of information and access to assistive devices are the main reasons for their lack of adoption. Lack of funding for upper limb assistive technologies has previously been identified as an issue that hampers their development [33].

Ease of doffing and donning and setup time affect the acceptance for any new device for upper extremity. On the basis of the findings of our study, ease of application and setup times appear to be important considerations for device development. Our finding contradicted a study by Liu et al [48] where the authors concluded that therapists’ effort expectancy was not a salient factor when adopting new technologies or devices. However, our findings are congruent with a study by Hughes et al [35] where the authors found that when developing a new device for the upper extremity, ease of setup and use was the most important factor identified by health care professionals and the second factor identified by the patients and their caregivers. Thus, for any future design of assistive devices for upper extremity, it is important to insure the ease of donning the device and setup time are considered.

### Limitations

Given the exploratory nature of this study, a number of limitations need to be acknowledged. There may be some issues with transferability given the nature of the study sample, which included 16 participants in total (8 people with stroke and 8 therapists) from one site. Although focus groups are more efficient than individual interviews, they have their own limitations, especially with heterogeneous groups. Moreover, despite efforts that were made to hear from everyone, some participant voices dominated the discussion. Furthermore, given time constraints within the focus groups, it was not always possible to clearly delineate the cause of all users’ concerns.

### Conclusions and Future Work

This exploratory study investigated perspectives from two different populations (ie, people with stroke and therapists) regarding available wearable robotic devices for the upper extremity. Participants with stroke had more knowledge about passive devices over active ones despite equivocal evidence about the efficacy of passive devices, whereas therapists had more knowledge about existing therapeutic devices. In general, participants’ experiences with available robotic devices for upper extremity were not positive because of multiple issues that included concerns about cost-effectiveness and concerns about the potential long-term loss of independence. Although participants supported the need to develop new robotic devices for the upper extremity, their needs were diverse.

This research lays the groundwork for a variety of future studies. This could include studies with a larger sample of participants representing more diverse age ranges, geographical locations, and patients with different neurological disorders (such as spinal cord injury or cerebral palsy patients) which could add to our study findings. Furthermore, future studies could investigate whether the gap between users’ current activity levels and their desired activity levels influences their preferences regarding new robotic devices.

## Appendix

Multimedia Appendix 1 Focus Group Interview Guide.
