# Supplementary material for: Perceptions of Existing Wearable Robotic Devices for Upper Extremity and Suggestions for Their Development: Findings From Therapists and People With Stroke
Source: JMIR Rehabil Assist Technol. 2018 May 15;5(1):e12. doi: 10.2196/rehab.9535 (PMC5974461; doi:10.2196/rehab.9535)
Supplement: Multimedia Appendix 1 [file rehab_v5i1e12_app1.pdf]

Outpatient Clinician Focus Group Guide

Intros, explanation of process and preliminary info.

- **Thank participants for coming.**
    - We invited you here today because you have more than a year experience working with either stroke survivors or older adults.
- **Introduce self and observer.**
    - I just want to remind you that the focus group will be tape recorded so I can go back and more thoroughly process what was discussed today. The information you provide is confidential and thus we will not be using your real names or identifying information without your permission. Observer is here to assist in moderating and taking some notes.

- **Ground Rules.**

    - In our discussion today, there aren't any right or wrong answers. We expect that each of you will have different points of view and we want to hear from everyone. Please feel free to share your opinions whether you agree or disagree with what someone else has said. As you are aware, we are recording the session today because we don't want to miss anything that anyone has to say. Your names will not be included in any reports, presentations, or publications that come out of our discussion today. Your comments are confidential to the extent allowed by law and we would like to ask all group members to let what is said in our group today stay here. This will protect everyone's privacy and help to make every feel comfortable to speak freely.

- **Explain focus group process and objectives.**
    - Today we would like to talk to you about your experiences in working with either older adults or stroke survivors.
    - Specifically, we aim to know your experience and your opinion regarding using wearable (robotic) devices for upper limb rehabilitation or compensation (assistance).
    - Robotic wearable devices do not replace therapists, but provide an additional means by which patients can engage in meaningful practice.
    - We are working with engineers from SFU to design a new device for upper extremity rehabilitation/assistance.
    - We imagine this device would be suitable for older adults and stroke survivors with mild to moderate impairment.
    - We are hoping to get your opinions about if and how this could be useful for your practice and what features you would like a device to have.

- **Info about client population.**
    - Before we begin, can we go around and state your name and how many years you have worked with either older adults or individuals with stroke.

Part 1 Questions: Previous experience in using wearable devices for upper limb rehabilitation/assistance.

1. **<u>Open-ended question</u>** Please tell me about your experience with wearable devices for upper limb rehabilitation/assistance.
    a. Tell me about the problems you faced in your practice?
    b. How significant was these problems?
    c. **<u>Probe</u>**: please give me an example from your experience about the significance of this problem.
    d. **<u>Prompt</u>**: We mentioned some devices that you used in practice, what do you don't like about this device/s?

<u>Part 2 Questions: Understanding clinician requirements about a new device for upper extremity rehabilitation/assistance.</u>

1. **<u>Open-ended question</u>** if you are to design a new device for upper extremity rehabilitation/compensation, what would be helpful for you?
    a. **<u>Probe:</u>** What do you think is still missing?
    b. **<u>Probe:</u>** What kind of devices would you like to see developed?
    c. **<u>Probe:</u>** Which population you would use this device with?
    d. **<u>prompt:</u>** In the population you mentioned would you recommend a device for rehabilitation or compensation.
    e. **<u>Probe:</u>** In this population, what joints of the upper limb are of great importance to work on?
2. **<u>What features you think this device should have?</u>**
    a. **<u>Prompt</u>**: What about portability?
    b. **<u>Prompt</u>**: Which kind of activation you would like to be used in this device? (i.e. EEG, EMG, etc.).
    c. **<u>Probe:</u>** Which data you want this device to record and save?
    d. **<u>Probe:</u>** please tell me more about the significance of the data you mentioned.
    e. **<u>Probe:</u>** As a clinician, how would you like to communicate with such a device? (i.e. manually, or via Bluetooth, using internet, etc.).

<u>Part 3 Questions: Integrating the device into practice.</u>

a. **<u>Open-ended question:</u>** When you think about this device (these devices), how would you use it in your clinical practice?
b. **<u>If you would use this device in rehabilitation.</u>** Can you think about rehabilitation protocols to use this device in?
c. **<u>Probe:</u>** How might you use it during a therapy session?
d. **<u>Probe:</u>** In this rehabilitation protocol, which type of user engagement you prefer? passive, active assistive or active resistive protocols?
e. **<u>Probe:</u>** Please tell me about the significance of the type of user engagement you preferred.
f. Which aspects you consider the most in this protocol? Number of repetitions, speed of the movement, or range of motion.
g. **<u>Probe:</u>** Please prioritize and tell your preference as to the first, second and the third and so on.

Part 4 Questions: Concerns about wearable robotic devices in these populations.

a. **Open-ended question:** What concerns you think in using this device (these devices) in practice?
b. What do you think about the appearance of th(is/ese) kind of device(s)?
c. **Probe:** Could you please tell me more about cosmetic concerns.
d. Is the device cost being a concern?
e. What do you think a reasonable cost for such device?
f. What do you think about the size and weight?
g. What would be a reasonable weight for such device?
h. If a compromise in weight/size/speed/power/robustness/portability is needed in terms of engineering aspects. What would you choose to compromise on?
i. Please prioritize and tell your preference as the first, second and the third and so on.

Part 5. Other question.

In this discussion, do you think we missed any point related to wearable robotic devices for upper extremity?

Outpatient Focus Group Guide (People with stroke)

Intros, explanation of process and preliminary info.

- **Thank participants for coming.**
  - We invited you here today because we want to find out your ideas about robotic assistance and rehabilitation for the upper extremity.
- **Introduce self and observer.**

- I just want to remind you that the focus group will be tape recorded so I can go back and more thoroughly process what was discussed today. The information you provide is confidential and thus we will not be using your real names or identifying information without your permission. Observer is here to assist in moderating and taking some notes.

- **Ground Rules.**

  - In our discussion today, there aren't any right or wrong answers. We expect that each of you will have different points of view and we want to hear from everyone. Please feel free to share your opinions whether you agree or disagree with what someone else has said. As you are aware, we are recording the session today because we don't want to miss anything that anyone has to say. Your names will not be included in any reports, presentations, or publications that come out of our discussion today. Your comments are confidential to the extent allowed by law and we would like to ask all group members to let what is said in our group today stay here. This will protect everyone's privacy and help to make every feel comfortable to speak freely.

- **Explain focus group process and objectives.**
  - We aim to know your experience and your opinion regarding using wearable (robotic) devices for upper limb rehabilitation or compensation (assistance).
  - Robotic wearable devices do not replace therapists, but provide an additional means by which patients can engage in meaningful practice.
  - We are working with engineers from SFU to design a new device for upper extremity rehabilitation/assistance.
  - We imagine this device would be suitable for older adults and stroke survivors with mild to moderate impairment.
  - We are hoping to get your opinions about if and how this could be useful for your practice and what features you would like a device to have.

- **Info about client population.**
  - Before we begin, can we go around and state your name and Anything you would like the group to know.

Part 1 Questions: Previous experience in using wearable devices for upper limb rehabilitation/assistance.

2. **Open-ended question** please tell me about your experience with wearable splints, slings, or other devices for upper limb rehabilitation/assistance.
   a. Tell me about the problems you faced?
   b. How significant was these problems?
   c. **Probe**: please give me an example from your experience about the significance of this problem.
   d. **Prompt**: We mentioned some devices that you used in practice, what do you don't like about this device/s?

Part 2 Questions: Understanding requirements about a new device for upper extremity rehabilitation/assistance.

3. **<u>Open-ended question</u>** if you were to have a new device for upper extremity rehabilitation/compensation, what would be helpful for you?
    a. **<u>Probe:</u>** What do you think is still missing?
    b. **<u>Probe:</u>** What kind of devices would you like to see developed, which joints they would be included?
    c. **<u>Prompt:</u>** you recommend these devices for rehabilitation or compensation.
    d. **<u>Probe:</u>** what joints of the upper limb are of great importance to work on?
4. **<u>What features you think this device should have?</u>**
    a. **<u>Prompt</u>**: What about portability?
    b. **<u>Prompt</u>**: how would like to trigger it? (i.e. EEG, EMG, etc.).
    c. **<u>Probe:</u>** Which information would this device to record and save?
    d. **<u>Probe:</u>** please tell me more about the significance of the information you mentioned.
    e. **<u>Probe:</u>** how would you like to communicate/interact with such a device? (i.e. manually, or via Bluetooth, using internet, etc.).

<u>Part 3 Questions: Integrating the device into life.</u>

- **h.** **<u>Open-ended question:</u>** When you think about this device (these devices), how would you use it/them?
- **i.** **<u>What would you think about using this device in rehabilitation?</u>** Can you think about rehabilitation protocols to use this device in?
- **j.** **<u>Probe:</u>** How might it be used during a therapy session?
- **k.** **<u>Probe:</u>** what is the type of user engagement you would prefer?
- **l.** Which aspects you consider? Number of repetitions, speed of the movement, or range of motion.
- **m.** **<u>Probe:</u>** Please prioritize and tell your preference as to the first, second and the third and so on.

<u>Part 4 Questions: Concerns about wearable robotic devices in these populations.</u>

- **j.** **<u>Open-ended question:</u>** What concerns you think in using this device (these devices)?
- **k.** What do you think about the appearance of th(is/ese) kind of device(s)?
- l. **<u>Probe:</u>** Could you please tell me more about cosmetic concerns.
- m. Is the device cost being a concern?
- n. What do you think a reasonable cost for such device?
- o. What do you think about the size and weight?
- p. What would be a reasonable weight for such device?
- q. If a compromise in weight/size/speed/power/robustness/portability is needed in terms of engineering aspects. What would you choose to compromise on?
- **r.** Please prioritize and tell your preference as the first, second and the third and so on.

<u>Part 5. Other question.</u>

In this discussion, do you think we missed any point related to wearable robotic devices for upper extremity?
